# Iodine deficiency and thyroid nodular pathology - epidemiological and cancer characteristics in different populations: Portugal and South Africa

**DOI:** 10.1186/s13104-015-1155-3

**Published:** 2015-07-01

**Authors:** José Eduardo Carvalho Santos, William John Kalk, Miguel Freitas, Isabel Marques Carreira, Miguel Castelo Branco

**Affiliations:** Faculty of Health Sciences, University of Beira Interior, Covilhã, Portugal; CICS-UBI Health Sciences Research Centre, Faculty of Health Sciences, University of Beira Interior, Covilhã, Portugal; Centro Hospitalar Cova da Beira, Covilhã, Portugal; Department of Endocrinology, Faculty of Health Sciences, University of the Witwatersrand, Johannesburg, South Africa; Department of Biostatistics, Faculty of Health Sciences, University of Beira Interior, Covilhã, Portugal; CIMAGO–Centre of Investigation in Environment, Genetics and Oncobiology, Coimbra, Portugal

**Keywords:** Thyroid, Goiter, Papillary carcinoma, Follicular carcinoma, Anaplastic carcinoma, Iodine deficiency, Iodine nutrition, Iodized salt, Seafood nutrition

## Abstract

**Background:**

The prevalence and pathology pattern of iodine deficiency (ID) related disorders are influenced by the dietary iodine intake: low iodine leads to thyroid nodular enlargement, to an increase in the incidence of thyroid cancer, an increase in anaplastic carcinomas and to an alteration in the papillary to follicular neoplasia ratio. This study aims at highlighting the effects of ID by comparatively evaluating the pattern of thyroid nodular pathology in different populations that, although geographically distant and heterogeneous, both had iodine deficiency at the time of data gathering and are at high altitude: Beira Interior (BI) in Portugal and Johannesburg (JHB) in South Africa. (S.A.) Mandatory salt iodization introduced in S. A. in 1995 has recently been shown to have resulted in the correction of ID.

**Methods:**

Evaluation of thyroid histology reports over a 6 year period in BI and a 5 year period in the JHB area.

**Results:**

Region of BI: 278 patients with histology reports-60 were malignancies (21.2 %): 31 papillary carcinomas, 22 follicular cancers (18 follicular carcinomas and 4 Hürthle cell tumours), 3 medullary carcinomas and 4 anaplastic carcinomas. Region of JHB: 136 histology reports- 33 were malignancies (24.3 %): 13 papillary carcinomas, 15 follicular cancers (10 follicular carcinomas and 5 Hürthle cell tumours), 1 medullary carcinoma, 3 anaplastic carcinomas and 1 metastatic carcinoma into the thyroid. There was an overlap in the frequencies of all histology types, of particular relevance in the relatively high anaplastic carcinoma incidences and in the papillary to follicular carcinoma ratios which was close to 1 in both areas- BI area ratio: 1.4 and JHB area ratio: 0.87, with overlapping 95 % CI’s, also confirmed by the results of the chi-square calculations.

**Conclusions:**

During the study periods evaluated both study areas displayed pathology patterns usually found in ID. Public information regarding the negative consequences of ID combined with the availability of affordable iodized salt are likely to achieve the goal of the elimination of ID. Sea based nutrition, (naturally iodine containing), may also contribute to the elimination of ID, particularly at times when salt restriction tends to be generally advised.

## Background

Iodine is the essential component of the thyroid hormones T3 and T4 [1], which regulate metabolic processes in most cells and play an important role in the early growth and development of most organs, particularly the brain [2]. Globally iodine deficiency (ID) is the most common preventable cause of brain damage, with more than 2 billion people from 130 countries at risk [3], including an estimated 241 million children of school age [4]. It is especially prevalent worldwide in inland continental or mountainous regions [1], in areas beyond high mountain ranges [5] and may be independent of sea proximity. Consequences of ID include variable degrees of intellectual impairment, with demonstrable neuropsychointellectual deficits [6, 7], compromised reproductive potential [8], development of goitre, thyroid nodular pathology [1] and an increase in the incidence of thyroid cancer [9, 10]. Low iodine intake leads to an adaptive process that results in sustained increased secretion of thyroid-stimulating hormone (TSH), which has a direct trophic effect on the thyroid gland leading to hypertrophy and hyperplasia [1] that progresses to nodular development [1], focal hyperplasia, adenoma formation, autonomous growth and even malignant transformation [11]. ID has been shown to be associated not only with an increase in thyroid cancer incidence rate but also with an alteration in the ratio of papillary to follicular (P:F) neoplasia, from the usual significant predominance of papillary carcinoma observed in iodine sufficiency [9]. The P:F thyroid cancer ratio is reported as ranging from 6.5:1 to 3.4:1 in areas of high iodine intake, and 1.7:1 to 0.19:1 in ID areas [12]. This ID pattern has been shown to be reversed when iodine deficiency is corrected [9].

South Africa is documented as having had areas of ID in the years before the introduction in 1995 of legislation requiring all manufactured salt to contain potassium iodate [13]. This policy has been shown successful in the elimination of ID in South Africa [14]. In Portugal salt iodisation is done on a voluntary basis, there being no national program for the elimination of ID and no regular Urinary Iodine (UI) monitoring [15]. There has been no general population data on iodine nutrition (INu) [4, 16] the only countrywide study available referring to the population group of pregnant women, demonstrating significant iodine deficiency throughout the country [17].

In population studies, measuring the median urinary iodine concentration (UIC) is the most accurate method of assessing and monitoring the INu status, through random spot urine samples measured in μg/L [18]. Iodine deficiency has been successfully corrected in S.A. [14] following the introduction of mandatory salt iodization in 1995 [13]. In the years preceding that, evaluation of INu using UIC was not easily nor routinely available. An alternative method of assessing INu through parameters relating to the thyroid histology (THist) pattern has been previously referred to and documented [9].

The aim of the present study is to evaluate the thyroid histology pattern of the population of the inland region of BI in Portugal and to compare it with the available histology pattern from the population of JHB, South Africa, prior to mandatory iodization of salt. These areas had in common: ID during the time periods evaluated [13, 17] and being high altitude regions, away from the coast. The consequences of inadequate iodine intake, the importance of adequate public information on this issue, salt iodization and of the need for consumption of sea food based nutrition are highlighted.

## Methods

### Study population

This study was approved by the ethics committees of the Faculty of Health Sciences of the University of Beira Interior and of Centro Hospitalar Cova da Beira (Hospitals of Covilhã and Fundão), as well as by the Committee for Research on Human Subjects of the University of the Witwatersrand/Baragwanath Academic Hospital.

The ultimate aim of the current study is to contribute towards the elimination of ID in countries and regions in which this problem is still prevalent, highlighting it’s serious negative consequences and proposing possible solutions that are easily applicable and affordable. Towards this aim, a comparative evaluation of the THist patterns from the population of the inland region of BI in Portugal and from the population from JHB, reflected by the predominantly black African population seen at the largest referral Hospital in S. A., Baragwanath Hospital (BH) was sought. The available thyroid histopathology pattern from the JHB, S.A. area, relating to a period prior to the introduction of mandatory salt iodization in 1995, was used [19].

The study area of BI, in Portugal is situated beyond the mountain range of Serra da Estrela, a mountainous range stretching across 115 km, with its highest peak at 1993 m above sea level [20]. The area of JHB is situated in a plateau at approximately 1750 m of altitude [21].

The introduction of mandatory salt iodization in South Africa [13] has been proven successful in eliminating ID [14]. Whilst this noteworthy success demonstrates an easy to follow example for regions that still experience the problem of ID it also means that UIC data demonstrating the previous ID status in South Africa would have had to be obtained from publications that preceded this measure. The evaluation of the histopathology pattern and its characteristics is an alternative and complementary way to measurements of UIC in demonstrating ID, as has been highlighted in previous studies [9].

### Diagnosis evaluation by histology

Patients that had histology available had been selected for thyroid surgery, in both areas, based on cytology criteria, ultrasound findings or clinical indications. These included family history of thyroid malignancy, an enlarging nodule or the presence of neck lymphadenopathy. A representative and illustrative sample of the thyroid nodular pathology in each of these areas was sought so as to enable a comparative evaluation of the patterns of pathology in these two areas.

Histology reports relating to patients residing in the inland region of BI, performed during the period between January 2002 and December 2008 were evaluated. The 6 year time period was arbitrarily chosen, aiming at obtaining a representative sample of the thyroid nodular pathology in the area. All reports from the local major referral Hospitals linked to the University of Beira Interior, (the only State University in this region), namely the Hospitals of Covilhã and Fundão, were obtained. This data was complemented with histology reports from the Portuguese Oncology Institutes of the three main nationwide centres of Lisbon, Oporto and Coimbra (the latter being the main Oncology referral centre for the region of BI), from patients that provided a residential address from the study area of BI.

Thyroid histology reports evaluated from the JHB area related to patients that had presented to the BH thyroid clinic in JHB during the 5 year period between January 1984 and December 1988 [19], this corresponding to a time preceding the introduction of mandatory salt iodation in South Africa [13].

The standard method for general histology with hematoxylin and eosin staining (H&E) was employed in both study areas, histology examinations having been performed by specialist pathologists with an interest in thyroid pathology at Academic Pathology Centres. The diagnostic criteria followed at both centres: CEDAP, linked to the University of Coimbra and at the South African Institute for Medical Research (S.A.I.M.R.), in Johannesburg, connected to the University of the Witwatersrand, followed the same diagnostic criteria, as defined by the W.H.O.

Although the time periods for data collection were different, they both correspond to periods when ID prevailed in each study area [13, 17].

### Statistical analysis

The thyroid pathology pattern is described in terms of absolute Frequencies and Percentages. The 95 % Confidence Intervals (CI) of percentages were calculated from the Normal distribution, except for small percentages where the exact probabilities from the binomial distribution were calculated [22]. The results obtained for BI and JHB were compared using the chi-square test, and its achieved power was determined using the obtained values of alpha, effect size and sample size (using G*Power) [23]. Differences between the relative frequencies of papillary cancer, follicular cancer and other thyroid neoplasias in JHB and BI were tested calculating the alpha and beta error of the chi-square test (G*Power). The 95 %CI’s of the papillary to follicular cancer ratios were calculated from the Normal distribution [22].

## Results

**Fig. 1:**
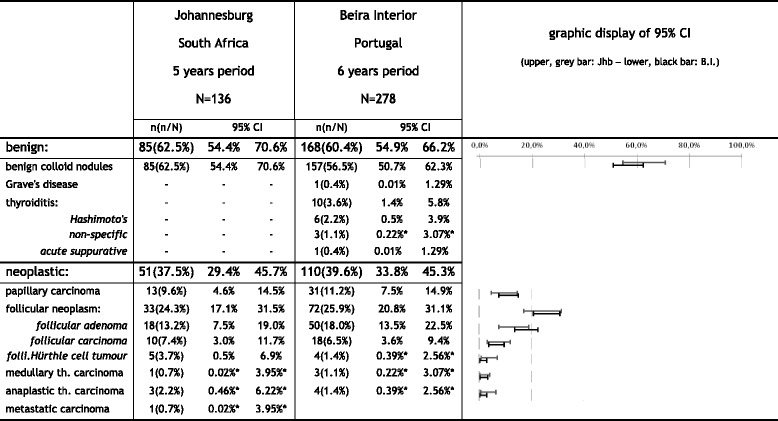
Frequency of different pathology types in the two study areas. *calculated using the exact probabilities of the Binomial distribution (np<5)

The histology results from the region of BI provided a total of 278 patient’s histological exams for evaluation. These were from 37 male patients and 241 female. Their ages ranged from 15 to 83 years of age (median 54 years). In the JHB area there was a total of 136 histological exams available, 12 from male patients and 124 from female. Their ages ranged from 18 to 74 years (median 51 years) [19]. The relative frequencies of the different pathology types in the two areas are displayed in Fig. 1, which also displays an overlap in the 95 %CI’s of all histological types. The chi-square (two-sided) α error probability was 0.606 and an achieved power (1 − β) of 0.9987 was obtained in a post-hoc calculation. The papillary to follicular carcinoma ratios were close to 1 in both areas: BI area ratio: 1.4 with 95 % CI from 0.816 to 2.434, and JHB area ratio: 0.87 with 95 %CI from 0.412 to 1.821. The obtained chi-square (df = 2) two-sided α error was 0.539 and the power of the test was 0.913 confirming the overlap shown in the 95 % CI.

**Fig. 2:**
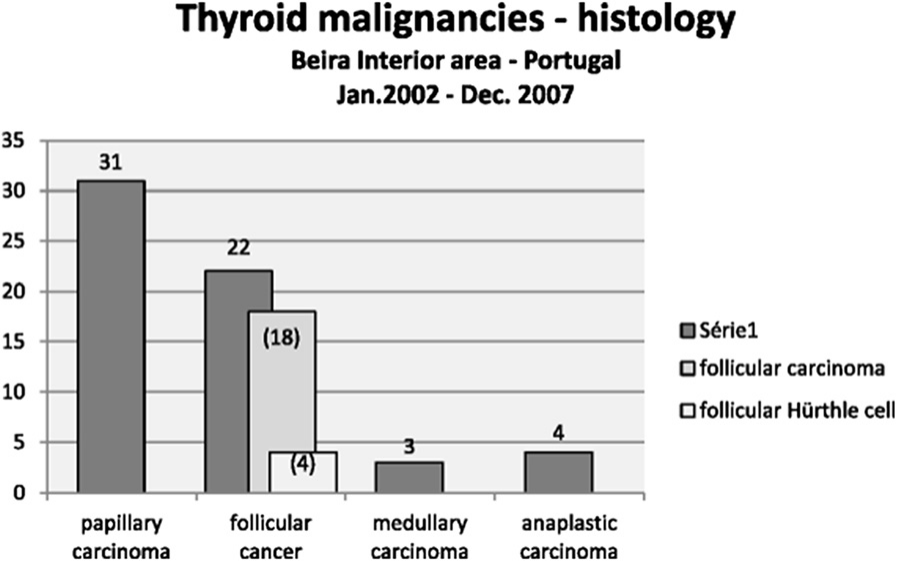
Histological diagnosis (illustrative display)—Thyroid malignancies—Beira Interior: 6 year period (January 2002–December 2007). The types of follicular cancer are discriminated

**Fig. 3:**
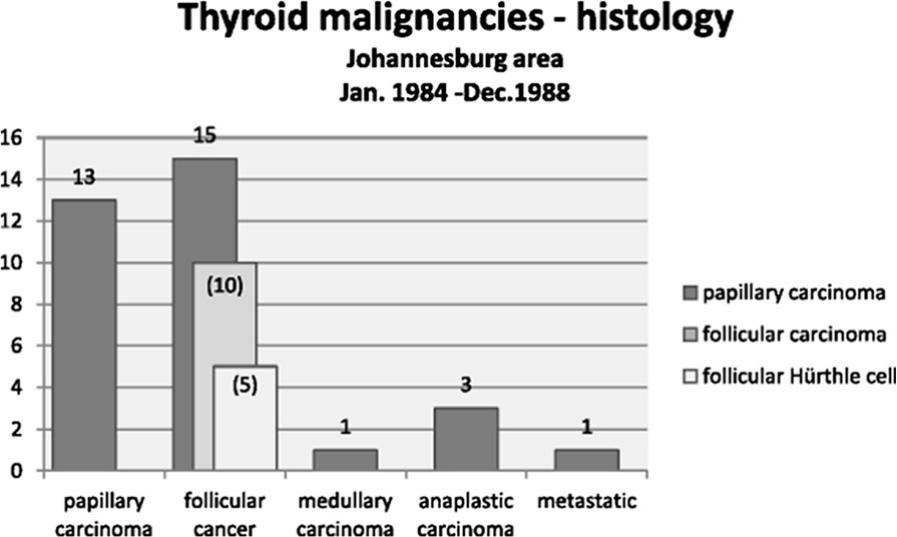
Histological diagnosis (illustrative display)—Thyroid malignancies—Johannesburg: 5 year period (January 1984–December 1988). The types of follicular cancer are discriminated

The analysis of the data from the Beira Interior region, in Portugal, revealed that 60 of the 278 patients had malignancies (21.2 %). These were made up of 31 papillary carcinomas, 22 follicular cancers (18 follicular carcinoma and 4 Hürthle cell tumour), 3 medullary carcinomas and 4 anaplastic carcinomas (Fig. 2). In the JHB area 33 of the total of 136 patients (24.3 %) had malignancies. These were made up of 13 papillary carcinomas, 15 follicular cancers (10 follicular carcinoma and 5 Hürthle cell tumour), 1 medullary thyroid carcinoma, 3 anaplastic thyroid carcinomas and 1 metastatic carcinoma into the thyroid (Fig. 3).

## Discussion

Iodine is broadly distributed in the environment in the form of iodide [1] and is concentrated in sea water and in marine life [5]. Under normal circumstances iodine is absorbed as iodide through the digestive tract mainly through the walls of the stomach and the small intestine [24], iodine deficiency usually resulting from inadequate iodine intake.

In Africa dietary ID is the major determinant of thyroid pathology, ID goitre being the most commonly observed thyroid disorder [25]. Substantiating evidence of the persistency of ID in Africa has been the predominance of follicular carcinoma for decades [25], as opposed to the usual papillary carcinoma predominance in iodine sufficient areas [9]. Recently, improvements in iodine intake led to reductions in goitre prevalence [25].

Baragwanath Hospital is the biggest Hospital in Africa and treats patients primarily from the JHB area. The 5 year histology evaluation in the JHB (BH) area in the present study (1984–1988), relates to a time when iodine deficiency was present in this region, before iodine supplementation legislation requiring all table salt manufactured in South Africa to contain potassium iodate was introduced in 1995 [13].

Evaluating the thyroid pathology data from both study areas (displayed in Fig. 1), showed coinciding values in the relative incidences of the different histology types as demonstrated by the overlapping 95 % CI’s of each thyroid neoplasia. This similarity of the thyroid malignancy patterns was also confirmed by the achieved power of the chi-square test.

The malignancy rate in the inland region of BI, in Portugal was 21.2 % (60 malignancies from a total of 278 patients) and in the JHB (BH) area during the 5 year evaluated period was 24.3 % (33 malignancies out of a total of 136 patients that undergone thyroid surgery).

The histology pattern displayed in both study areas was indicative of ID [9], particularly regarding the papillary to follicular carcinoma ratios, that were close to 1 in both areas, and the relatively high anaplastic thyroid carcinoma incidences: BI area papillary/follicular ratio of 1.4 (95 % CI: 0.816 to 2.434), with a total of 4 anaplastic carcinomas out of a total of 60 malignancies (6.7 %); JHB area papillary/follicular ratio of 0.87 (95 % CI: 0.412 to 1.821) and a total of 3 anaplastic carcinomas out of a total of 33 malignancies (9.1 %). The malignancy rates reflect the selection criteria for surgery based on cytology results, as well as the existing ID in both areas in the study period.

Statistics relating to the economically depressed study region of BI are probably underrepresented, considering that approximately 30 % of the population in this area are over the age of 65 years, (60,260 of a population of 211,205) [26] whose offspring tend to migrate to the more affluent coastal metropolitan areas of the country, where they work and live. Should these elderly patients require surgery, particularly if the possibility of cancer is mentioned, they would tend to have it in the metropolitan residential areas of their younger relatives, being statistically lost from their usual area of residence in BI.

Voluntary salt iodization at a low iodine concentration was not successful in eliminating ID in SA before the introduction of mandatory salt iodization at a higher iodine concentration in 1995 [13]. This measure has succeeded in the elimination of ID throughout South Africa [14].

China successfully eliminated ID using a coordinated approach which started in 1978 with the ruling that the cost of iodization of salt be borne by the state, as this would be to the benefit of the entire country’s population [27].

In Portugal, where the existing legislation relating to salt iodization was adopted in 1969 on a voluntary basis [15], there is still a lack of national general population data regarding INu [15, 16], no national committee existing for monitoring the elimination of ID [15]. This voluntary salt iodization legislation has been shown ineffective in the only countrywide published study on INu, done in the selected population group of Portuguese pregnant women, which demonstrated inadequate iodine intake throughout Portugal [17], with a nationwide median UIC value was 82 · 5 μg/L. The region of BI was the most iodine deficient area of Continental Portugal with a median UIC of 67 · 6 μg/L. Illustrating that INu is determined by the amount of iodine ingested and not necessarily the proximity to the sea, the mid Atlantic islands of Açores had an even lower median UIC of 50 · 0 μg/L [17]. Two additional, complementary studies on INu in Portugal were recently published, (in Portuguese, English Abstract only), done on schoolchildren aged 6–12, from 78 schools from the region of Continental European Portugal [28] and from the regions of the Atlantic Islands of Madeira and Açores (school children and pregnant women aged 17–46 years) [29], displaying results that could be considered in keeping with the only nationwide study available, previously cited [17]. These three recent studies [17, 28, 29] and the influence exerted by the working group on Thyroid Studies of the Portuguese Endocrine Society, led to the publication of “Guidelines” in August 2013 [30], by the Directorate General of Health, recommending the prescription of a daily supplement of potassium iodide in a dose of 150 to 200 μg to pregnant, breastfeeding or pre-pregnancy age women. Whilst these developments are positive in attempting to eliminate ID, the issuing of guidelines, without supporting legislation or the combination of other complementary measures (adequate public information regarding the consequences of ID) may well fail to produce the expected results. The effectiveness of these guidelines still has to be proven.

The successful correction of ID requires easy and affordable access to iodized salt by the general population. The implementation in China, in 1978, of the policy that the cost of salt iodization should be borne by the Government, combined with adequate transportation, packaging and distribution measures ensured the availability and quality control throughout the vast country. This contributed to the success in the elimination of ID [27, 31]. These measures recognized the benefits to the entire population and to the country of access to iodized salt. China’s successful strategies in the elimination of IDD were achieved through the combination of carefully implemented salt iodization programs combined with on-going sub-national population monitoring for iodine nutrition [31]. Its success made it an example to be followed.

In South Africa the price of iodized salt is the same as for non iodized salt and both have been generally available countrywide, even before the introduction in 1995 of mandatory salt iodization at a higher level than before. By contrast, in Portugal iodized salt (with specification of iodine content) is generally not available. The only brand available is priced at more than double that of non-iodized salt (unpublished results). Since Portugal has salt production facilities [15] legislation requiring all commercially available salt to have a set amount of potassium iodide, as was done in South Africa in 1995 [13], could easily be implemented. To achieve the aim of eliminating ID adequate public information highlighting the serious adverse effects of iodine deficiency could provide the motivation for people to adopt a pro-active stand regarding the use iodized salt. Additionally, promoting the consumption of sea generated food products, naturally iodine containing, would be an important complementary measure, especially at times when reducing daily salt intake is now generally recommended.

## Conclusions

The study regions evaluated of BI in Portugal and JHB in South Africa display patterns of thyroid nodular pathology that were shown to be similar in both study populations, during the periods evaluated, indicative of ID as evidenced particularly by the papillary to follicular carcinoma ratios (close to 1 in both study areas) and the relatively high number of anaplastic carcinomas [9, 12]. This in spite of the differences in geographical location, the racial and ethnic composition of the population samples, as well as the different time periods of evaluation.

The example of South Africa’s success in changing from having ID in the past to its current iodine sufficiency status shows that the elimination of ID is achievable provided there is easy and affordable access to iodized salt and especially if the population is adequately informed as to the serious negative consequences of iodine deficiency. Promoting sea based nutrition could also be important.

These measures could be applicable, as public health measures, to other populations in different parts of the world still experiencing the problem of ID.

## Abbreviations

BH: Baragwanath hospital
BI: Beira interior
CI: Confidence intervals
H&E: Hematoxilin and eosine
ID: Iodine deficiency
IDD: Iodine deficiency disorders
INu: Iodine nutrition
JHB: Johannesburg
SA: South Africa
TSH: Thyroid stimulating hormone
T3: Tri-iodothyronine
T4: Thyroxine
THist: Thyroid histology
UIC: Urinary iodine concentration
WHO: World Health Organization
